# Efficacy of Age-Specific High-Intensity Stretch-Shortening Contractions in Reversing Dynapenia, Sarcopenia, and Loss of Skeletal Muscle Quality

**DOI:** 10.3390/jfmk3020036

**Published:** 2018-06-19

**Authors:** Brent A. Baker

**Affiliations:** Health Effects Laboratory Division, Toxicology and Molecular Biology Branch, Systems Mechanophysiology and Aging Research Team, Centers for Disease Control and Prevention, National Institute for Occupational Safety and Health, Morgantown, WV 26505, USA;

**Keywords:** aging, stretch-shortening contractions (SSCs), resistance-type exercise training, dynapenia, sarcopenia, skeletal muscle quality, health-span

## Abstract

During the aging process, skeletal muscle performance and physiology undergoes alterations leading to decrements in functional capacity, health-span, and independence. Background: The utility and implementation of age-specific exercise is a paramount research agenda focusing on ameliorating the loss of both skeletal muscle performance and physiology; yet, to date, no consensus exists as to the most appropriate mechanical loading protocol design or overall exercise prescription that best meets this need. Thus, the purpose of this review is to highlight the most optimal type of exercise presently available and provide the most current, evidence-based findings for its efficacy. The hypothesis that high-intensity, stretch-shortening contractions (SSCs)—a form of “resistance-type exercise” training—present as the preferred exercise mode for serving as an intervention-based modality to attenuate dynapenia, sarcopenia, and decreased muscle quality with aging, even restoring the overall youthful phenotype, will be demonstrated. Conclusions: Appreciating the fundamental evidence supporting the use of high-intensity SSCs in positively impacting aging skeletal muscle’s responsivity and their use as a specific and sensitive countermeasure is crucial. Moreover, from an applied perspective, SSCs may improve skeletal muscle quality and rejuvenate health-span and, ultimately, lead to augmented functional capacity, independence, and quality of life concomitant with decreased morbidity.

## 1. Introduction

Aging is a shared biological process that occurs in vivo at the organism level; although many species tend to age at varying rates, ultimately it is defined as the chronological phenomenon that is concurrent with alterations in the underlying biology. Further, aging is associated with numerous conditions that compromise health, independence, and quality of life [1]. Musculoskeletal disorders (MSDs), which include a spectrum of conditions that range from acute strains and sprains to chronic conditions such as maladaptation, overexertion, and repetitive use, are multifactorial conditions that can affect all individuals and present as a significant burden in terms of prevalence as well as fiscal impact [2,3]. Specific MSD concerns that present with the aging process in humans, as well as all mammals, are dynapenia, the age-related loss of strength specifically [4,5] (and loss of overall functional performance in general); sarcopenia, the age-related loss of skeletal muscle mass [6–8]; and decreased skeletal muscle quality (skeletal muscle performance per unit skeletal muscle mass). Interestingly, recent fundamental as well as translational data suggests that muscle quality may be the most specific and sensitive outcome metric pertinent to overall quality of life and longevity [9,10]. As these are familiar occurrences that may lead to decreased health-span (i.e., the period of life free from disability, morbidity, and chronic disease) [7], it is imperative that interventions specifically targeting and countering these age-related events are emphasized. Amongst common daily activities incurred by the neuro-musculo-skeletal system are skeletal muscle contractions that span a spectrum of intensities (i.e., low to moderate to high). Different modes of activities including, but not limited to, physical labor in occupational and military settings, competition in athletic arenas, and exercise in wellness and strength and conditioning domains that include both aerobic (i.e., biking, running, etc.) and anaerobic (i.e., resistance exercise, plyometric exercise, etc.) exercise are known to utilize stretch-shortening contractions (SSCs). SSCs are common muscular movements utilized by all mammalian species which include a successive pre-activation phase consisting of an isometric contraction, a stretch phase, and a shortening phase [11–13]. For these reasons, the current manuscript will emphasize the efficacy of age-specific, high-intensity SSCs’ potential for reversing the loss of muscular performance, skeletal muscle mass, and skeletal muscle quality that are accepted detrimental outcomes of MSDs that occur with the aging process, along with the supposition that SSCs can positively affect the overall aging skeletal muscle phenotype by restoring it to a more youthful state.

## 2. Aging

Aging itself is not considered a disease [14]; however, concurrent with the aging process is an increased susceptibility to various acute and chronic disease states, which is associated with the progressive decline in biomechanical and physiological function that potentially leads to dysfunction, disability, loss of independence, and, ultimately, death [15]. These concepts are important given that recent data show that by the year 2050, 25% of the world population will be 65 years of age or older, and certain areas of the world will have populations exceeding 40% of 65 years of age and older [16]. Furthermore, additional population data suggests that the number of individuals 80 years of age and older will triple during this time [17]. In the United States alone, it is estimated that by 2050, the number of individuals 65 years of age and older will double [18], while the domestic workforce’s current composition of 1-in-5 workers over the age of 55 is anticipated to be 1-in-4 workers over the age of 55 by 2020 [19]. Given that this robust level of population aging will impact most nations worldwide [16], a significant socio-economic challenge in terms of fiscal commitment has been incurred and will continue to progress. Overall, this is an exceptional burden, as such demographic shifts impact society as a whole; thus, due to these challenges being all-encompassing, the need for a systematic approach that not only sustains but enhances health-span is essential.

The syndrome that is aging is multi-factorial, integrated, and comprehensive in that it spans biological/physical, psychological/cognitive, and social domains, as well as being influenced by cultural and multi-generational environments that are dynamic and adaptive concepts. The concentration of this contribution will focus on the biological and physical concepts that present with and are impacted by aging; and, moreover, propose the most plausible intervention(s) that target functional and physiological decline.

## 3. Musculoskeletal Disorders (MSDs): Dynapenia, Sarcopenia, and Skeletal Muscle Quality

MSDs are defined by various agencies and governing bodies differently, given that the term is all-encompassing and intended to capture a group of diagnosable medical conditions that are quite distinct and present with various etiologies. As defined by the United States Department of Labor’s Bureau of Labor Statistics (BLS), MSDs include “cases where the nature of the injury or illness is pinched nerve; herniated disc; meniscus tear; sprains, strains, tears; hernia (traumatic and nontraumatic); pain, swelling, and numbness; carpal or tarsal tunnel syndrome; Raynaud’s syndrome or phenomenon; musculoskeletal system and connective tissue diseases and disorders, when the event or exposure leading to the injury or illness is overexertion and bodily reaction, unspecified; overexertion involving outside sources; repetitive motion involving microtasks; other and multiple exertions or bodily reactions; and rubbed, abraded, or jarred by vibration” [20].

As can be surmised by the breadth of the description of MSDs by the BLS nomenclature, the spectrum of disorders, conditions, and diseases are numerous (i.e., ranging from overt soft-tissue injury to overexertion and repetitive use events that may lead to the maladaptation of soft tissue without overt injury) and, indeed, include those states where alterations (both in function and physiology) are effected [21,22]. MSDs such as soft-tissue strain/sprain injury that result from mechanical/physical loading of soft-tissue are clinically diagnosable injuries/disorders and can result following exposure in any environment and at any age. These examples are initiated by focal mechanical/physical insult that induces overt injury [23–25] to the underlying micro-level structures such as sarcomeres [26], the excitation-contraction coupling system [27] and the surrounding interstitium [28]. Additionally, following the initial induction of injury, there is a putative secondary injury phase comprising the biological response that includes significant gradations of edema (i.e., swelling), inflammation, degeneration, regeneration, and repair and remodeling [29–34]. However, this phenomenon is very distinct from cases where maladaptation results, such is the case in physical work and exercise-induced events, and is accompanied by transient soreness and discomfort following loading of soft tissue [12,35,36]. These latter disorders have been reported in both humans [37] and animals [13,38] and typically present with no known etiology, while representing cases where functional loss occurs in the absence of any underlying obvious pathophysiology (i.e., overt inflammation, degeneration, etc.). Obviously, both overt skeletal muscle injury as well as maladaptation may occur at any age [35]; yet, specifically, with respect to maladaptive events incurred during aging, the conditions known as dynapenia and sarcopenia as well as skeletal muscle quality will be examined in this context.

Dynapenia or the age-related loss of skeletal muscle performance, specifically skeletal muscle strength and power [4,5], is a specific and sensitive biomechanical variable (i.e., biomarker) that has immense clinical significance. Loss of skeletal muscle performance historically has been believed to be caused by the loss of motor neuron innervation, the loss of the number of skeletal muscle fibers, and/or skeletal muscle atrophy that occurs with the aging process. However, more contemporary findings suggest that this association may be influenced at multiple levels and is not as straight-forward and direct as once believed [39].

Sarcopenia is defined as the age-related loss of skeletal muscle mass [6] and is associated with loss of functional capacity, frailty, decreased quality of life, self-sufficiency, and mortality [40,41]. This loss of skeletal muscle mass has been reported to decrease at a rate of ~10% per decade starting approximately at 50 years of age and is suggested to impact various other aging conditions such as frailty, cachexia, and loss of independence [42,43]. Indeed, it has been reported that, even in individuals that are otherwise considered “healthy”, skeletal muscle mass has been shown to decrease ~ 1% per year from its peak occurrence between the ages of 20–30 years old [44]. The loss of skeletal muscle mass is multi-factorial and many factors have been identified as contributors to this event; even though this is not an exhaustive list, a few of these factors are anabolic resistance [45–47], apoptosis [48,49], chronic inflammatory signaling associated with long-term, low-grade inflammation [50–53], and neuromuscular alterations such as motor unit loss that leads to skeletal muscle fiber denervation [45–47]. This latter event may be the most consequential, given that it could be thought of in terms of an explicit functional event: loss of motor units results in denervation and loss of skeletal muscle fibers, specifically the fastest skeletal muscle fibers, and finally loss of skeletal muscle function [54]. However, it should be noted that there are encouraging data suggesting that the modification of this fast-twitch skeletal muscle fiber population may be achievable, given the correct exposure stimulus [55]. Thus, more age-specific and -sensitive approaches should continue to be explored that will have a positive or optimal effect. However, even though this is the case, the loss of skeletal muscle mass as well as skeletal muscle performance and skeletal muscle quality is inextricably linked to the aging process; moreover, how each of these factors impact the overall aging process as well as being altered by aging is still not well understood.

From a functional or performance-based perspective, elements such as skeletal muscle strength and power continue to be the primary outcome focus [56,57] as well as skeletal muscle mass [44,58]. Further, physiological assessments have primarily utilized skeletal muscle mass as a primary outcome metric for improving function in aged populations, yet results continue to be equivocal. More recently, muscle quality, defined as skeletal muscle performance per unit muscle mass, has been suggested and supported by data from basic, applied, and epidemiological research as being a more specific and sensitive outcome metric with respect to aging (and with respect to exercise training as well) [9,59,60].

## 4. SSCs as “Resistance-Type Exercise” Training (RTET)

Physical activity in general and physical work and exercise (which are subsets of physical activity and may be defined as planned, structured, repetitive bodily movements aimed at enhancing various components of fitness [61,62]) specifically are essentially exposure–response (or dose-response) relationships that describe a change in the organism imparted by a stressor, in this case a physical stressor. Exposure–response events may be acute or chronic, with the latter being the case for most work and exercise occurrences insofar as we as individuals incur years of occupational employment as well as undertaking an exercise regime with adherence to this with months to years of chronic training. Although exposure–response (as well as dose-response) relationships continue to be a principal concern in fields such as quantitative risk assessment and have historically focused on other types of stressors such as chemical and biological agents, physical exposure–response investigations and data are severely lacking. Understanding how a physical exposure such as repeated work or exercise results not only in a response but in an optimal response across the lifespan of the organism is essential. This relationship may afford researchers, clinicians, wellness professionals, and employers the most effective information to formulate evidence-based and effective environments that promote age-specific, ideal exposures ultimately promoting optimized individual responses.

As physical activity, work, and exercise are well-studied events, it is skeletal muscle contractions (i.e., isometric, lengthening, and shortening contractions) which produce bodily movement and increased metabolism and energetic expenditure. It should be mentioned at the fundamental level that these skeletal muscle contractions typically have been investigated using isolated preparations; although there are limitations to the physiological translation of these types of models and investigations, they have produced a wealth of essential data [63–65]. In addition, there are groups, including ours, that implement skeletal muscle contractions in a model that is more physiologically relevant in that it combines all types of skeletal muscle contractions as they are conducted and utilized in vivo: this scenario is known as either SSCs [12,13,66–68] or a subcategory of the SSCs defined as the stretch-shortening cycle [11,69–72]. The nuance and distinction between these two independently combined contraction paradigms is that as the SSCs include and incorporate the sequential pre-activation of the isometric contraction followed by successive lengthening and shortening contractions, and these SSCs are incurred during all activities of daily movement in mammals [50]. The stretch-shortening cycle, on the other hand, is a very specific type of SSC, due to the nature of the elicited “stretch-reflex” that is predominantly focused on taking advantage of the important role of force potentiation [11]. Nevertheless, all types of skeletal muscle contractions may occur across a continuum of intensities—low to moderate to high—with high-intensity skeletal muscle contraction stimuli being the least experienced by aging skeletal muscles over this time period. Undeniably, it is the skeletal muscle contractions and the activities that coincide with this type of stimulus that are of the high-intensity nature, which is predominantly neglected as we age. High-intensity exercise training, specifically “resistance-type exercise” training (RTET) that typically is conducted at intensities ≥ 70–85% of one repetition maximum, has been recommended for enhancing skeletal muscle mass and strength in healthy and older individuals [73–76]. While there may be exercise prescription concerns for individuals with specific risk factors and those with comorbidities, prescribing this mode of exercise and the overall execution of these high-intensity skeletal muscle contractions are not contraindications by themselves; however, medical and professional oversight is warranted and advised for aged populations, as is with all populations undergoing this type of resistance exercise training.

## 5. Efficacy of SSC RTET in Restoring the Youthful Phenotype

Of all recognized modalities that may be utilized for the prevention and/or intervention of aging, exercise is the most accessible, effective, and multifactorial modality known to improve health and treat chronic disease [77,78]. Interestingly, it is high-intensity, strenuous exercise that has been reported to be effective for treating numerous chronic diseases ranging from diabetes and heart disease to cerebrovascular and pulmonary disease [77,79] and may even be a more favorable intervention compared to pharmacological agents in specific cases such as stroke [80]. The interest in exercise as a means for prevention and/or intervention is not a new concept; however, more recently, interest in its efficacy and evidence for its pluripotency has led to the now global initiative known as “Exercise is Medicine^®^ (EIM)” from leading agencies, associations, and centers such as the American College of Sports Medicine, the American Medical Association, and the Centers for Disease Control, as well as enjoying support from the Surgeon General of the United States. This initiative aims to incorporate physical activity assessment and exercise prescription into disease prevention and to translate evidence-based, scientifically-proven health benefits of exercise into the current and future healthcare system [78]. It is well-accepted that regular physical exercise, which includes resistance exercise loading, provides the most conclusive evidence for preserving, and even restoring, function and skeletal muscle mass with aging [7,81–85]. It is chronic resistance exercise loading, or training, that is unique in promoting restoration or improvements in skeletal muscle performance (i.e., strength, work capacity, fatigue resistance, etc.) and skeletal muscle mass [83,84], as evidence for these findings exist in both human [57,71,83] and animal studies [66]; yet, little evidence exists for improving skeletal muscle quality in various populations, including aged populations [7,59]. There are numerous human studies that have suggested that resistance exercise training is beneficial in countering distinct components of musculoskeletal health and aging [71,83,84]; however, no translational study has shown that resistance exercise training can restore musculoskeletal health with aging to youthful states entirely. The limitations of human intervention studies are noted in that small populations, subject adherence, and lack of overall control with respect to the biomechanical loading parameters is customary. For these reasons and with these limitations in mind, fundamental, in vivo animal models that are valid and representative of the human exposure–response relationship with respect to aging and exercise are an essential tool for investigating and quantifying these phenomena due to the exquisite control they allow over the biomechanical loading exposure as well as providing a means to evaluate, characterize, and quantify the integrated skeletal muscle response systematically. Interestingly, only recently have investigations begun to question and quantify the impact age-specific RTET imparts on skeletal muscle quality and whether this specific and sensitive outcome metric (i.e., “biomarker”) is modifiable with aging [13,38,66,86]. Initial studies by Cutlip and colleagues provided a basis for investigating non-injurious SSC RTET exposure in a validated, fundamental rodent model of aging, which quantified the adaptive and maladaptive response in young and old skeletal muscle, respectively [13]. These reports provided evidence that a general SSC RTET employed 3 days per week over a 1-month period repeatedly resulted in skeletal muscle performance decrements with no improvements in skeletal muscle quality in old rodents (i.e., 30 months of age). More recently, Rader and colleagues have conducted follow-up work that extended from those initial findings and have reported that when SSC RTET was implemented in old rodents that included both the 3 days per week RTET group and compared this with a 2 days per week RTET group over a 1-month period, the old rodents that underwent 2 days per week RTET adapted and were indistinguishable from their younger counterparts and, notably, had their skeletal muscle quality restored to that of a youthful state [66]. In a subsequent investigation from this same group, both the mode (i.e., isometric contractions alone, utilized as a minimalistic RTET approach) as well as repetition number was studied. With regards to both the utilization of isometric contractions in age-dependent RTET and/or reducing the number of SSC repetitions (i.e., compared with the previous stated study), neither the isometric contraction RTET nor the reduced SSC repetition protocols were successful in restoring skeletal muscle performance, skeletal muscle mass, and skeletal muscle quality [86]. In these fundamental in vivo studies, it has been established that skeletal muscle quality is modifiable with increasing age; yet, these data suggest that the RTET prescription or mechanical loading paradigm must be precisely prescribed in order to achieve these benefits (i.e., increased function, skeletal muscle mass, skeletal muscle quality, etc.) for all outcomes predicted, particularly in aged populations. Also, these studies emphasize that there is a very narrow “window” involving both the specificity and sensitivity of these physical/mechanical loading variables with respect to an RTET exposure–response relationship with aging, and that particularly frequency of exposure, rather than mode or repetition number, may be the more precise variable for consideration when designing and implementing age-specific RTET in aged populations [66,85] when skeletal muscle performance, skeletal muscle mass, and skeletal muscle quality are the focal outcomes for positively affecting, and restoring, health-span. Thus, when considered in total, the fundamental evidence from SSC RTET suggests that the utility of this mode of exercise is efficacious and applicable for translational approaches in aging populations. Importantly, these studies continue to emphasize that the understanding and discernment of the biomechanical loading envelope (i.e., the mechanical factors responsible for the exercise or protocol “prescription”) is imperative, just as is the intimate knowledge and expertise required for the dosing of a pharmacological agent when prescribed for a particular pathophysiological condition, disorder, or disease.

## 6. Conclusions

MSDs continue to be one of the foremost health concerns for detrimentally impacting health-span and quality of life across the aging continuum. MSDs are pervasive and affect all populations irrespective of age, occupation, race, gender, ethnicity, economic, or social status. These events persist even though our knowledge, understanding, public health system, medical expertise, and therapeutic rehabilitation are at an exceptionally high level. However, in total, MSDs continue to be one of the leading health and fiscal concerns domestically and in the developed world [2,3]. Dynapenia, sarcopenia, and skeletal muscle quality present as significant MSDs affecting most aging populations and, for a world population that is shifting to a predominantly older demographic, this is of immediate and significant concern. For these reasons, the current manuscript has provided succinct evidence as to the efficacy of age-specific, high-intensity SSC RTET as a potential countermeasure for reversing the loss of muscular performance, skeletal muscle mass, and skeletal muscle quality which occurs with the aging process and supports the supposition that SSC RTET can positively affect the overall aging skeletal muscle phenotype by restoring it to a more youthful status.
